# Opening the Workplace After COVID-19: What Lessons Can be Learned from Return-to-Work Research?

**DOI:** 10.1007/s10926-020-09908-9

**Published:** 2020-06-19

**Authors:** William S. Shaw, Chris J. Main, Patricia A. Findley, Alex Collie, Vicki L. Kristman, Douglas P. Gross

**Affiliations:** 1 University of Connecticut School of Medicine, 263 Farmington Ave, Farmington, CT 06030 USA; 2 Keele University, Keele, North Staffordshire ST5 5BG UK; 3 Rutgers, the State University of New Jersey, 120 Albany Street, New Brunswick, NJ USA; 4 School of Public Health and Preventive Medicine, Monash University, 553 St Kilda Road, Melbourne, VIC 3004 Australia; 5 EPID@Work Research Institute and Department of Health Sciences, Lakehead University, 955 Oliver Road, Thunder Bay, ON P7B 5E1 Canada; 6 University of Alberta, 2-50 Corbett Hall, Edmonton, AB T6G 2G4 Canada

The on-going COVID-19 crisis has had an unprecedented effect on workplaces across the globe. The extent of viral infection, illness, and fatalities has transformed or closed many workplaces and resulted in large numbers of temporarily furloughed or unemployed workers. Those most susceptible to the virus and its effects are the elderly or medically vulnerable, but physical distancing, stay-at-home orders, and isolation have produced drastic social, economic and health consequences for workers of all ages, with a disproportionate impact on those more disadvantaged. Some businesses and workplaces are beginning to reopen, albeit under extraordinary rules pertaining to physical distancing, personal protective equipment, and physical guards. The efficacy of such measures in the workplace are unknown, and we have much to learn about how workers adapt and function under these circumstances.

Some of the challenges of inviting workers back to the workplace mirror some of the issues that we recognize as commonplace in the return-to-work and occupational rehabilitation literature—the idiosyncratic nature of health and work, individual disease vulnerability, susceptibility to environmental hazards, the need for job flexibility and modification, and differences in workstyle, social capital, and organizational support. A recurring theme in the work disability literature is the heterogeneity of return-to-work outcomes for workers with a wide range of injuries, illnesses, and medical procedures (e.g., cardiac arrest, major trauma) [1, 2]. Within medical conditions, this variation has been attributed to demographic and health variables (age, fitness, health status, anthropometry), to workplace factors (e.g., supervisor support, ability to accommodate, physical demands), to psychological factors (e.g., perceived impairment, job stress, coping, fears of re-injury or worsening health conditions, catastrophizing), and to social factors (e.g., family caregiving roles, social support, economic factors) [3–7]. The COVID-19 workplace opening process may also need to address this complexity of factors.

## Worker Factors

Just as injury and illness have variable effects on workability, the COVID-19 crisis is likely to impact workers differently because of issues like threat of viral infection, health vulnerability, organizational perceptions, income levels, and seniority/job tenure. Perhaps we can learn from studies in occupational rehabilitation [3–7] that have demonstrated how job stress, depressed feelings, job dissatisfaction, fears of injury or retaliation, catastrophizing, perceived incivility, and other factors can complicate rehabilitation and recovery.

The COVID-19 crisis has created a new workplace hazard that will be a significant source of stress and anxiety for many workers. This is especially true where infection risks are greatest, where workers are deemed essential to continue working, and for workers who are particularly vulnerable. Opening of workplaces during COVID-19 is occurring against a backdrop of heightened levels of psychological distress in the community that crosses all sociodemographic divides. Distress may result from increased personal financial pressure, social isolation, fear of infection, or the threat of job loss. Returning to an uncertain working environment presents an additional stressor that will further affect the mental health of workers [8].

Workers who experience COVID-19 symptoms and return to work after a period of illness and quarantine may experience fatigue, anxiety, and/or reduced work tolerance [9]. They may face difficulties in access/travel to work, restrictions in social contact with others, and new training, equipment, or responsibilities. The social stigma associated with a COVID-19 diagnosis may alter social relationships and access to or interactions with colleagues. It is unclear how conjoint work that necessitates close physical proximity will be managed, though it seems that mandatory physical distancing will be a condition for workplace opening. The social support of longstanding colleagues may fracture, and it may be difficult or impossible to work side-by-side with peers for any prolonged duration. The workplace has never had such seismic shifts at a global level.

One concern is that workers who have been away from physically demanding work for several months may experience deconditioning that poses risks upon returning to work. In occupational rehabilitation after injury, workers build tolerance for work gradually before resuming heavy physical work demands. The same opportunity may not be possible after a COVID-related layoff, but workers should re-engage with work tasks gradually to allow re-adaptation to heavy loading. In addition, domestic pressures arising from the health or risk of ill-health in families can have a significant effect on workers’ wellbeing, sleep, and mental health, with possible increases in presenteeism and work absence. The most prevalent of all will be fears of novel coronavirus itself, ironically a consequence of the strong public messaging that underpinned the initial lockdown. This new fear of unseen infection hazards in the workplace, rather than hazards of work itself, will be difficult to manage using traditional safety training and disability management strategies.

## Workplace Factors

The COVID-19 crisis has led to an unprecedented need for employers to provide flexibility and leeway so their workers can continue to work productively from home, adopt different work habits, or work in a new or rapidly changing environment. From the occupational rehabilitation literature, we know that workers are highly variable in their need for job modification after injuries. Similarly, workers will have substantially different needs for job modification related to COVID-19. Supervisors will be an important resource for information and individual worker problem solving. Authors have commented on the critical role of immediate supervisors for effective implementation of proactive employer policies and practices in workplace safety and return-to-work [10]. With COVID-19, workers will rely heavily on immediate supervisors to interpret the policies and practices of owners and corporations. In providing support and guidance, managers will be asked to address a wide range of effects not only of the virus, but of the impact of physical distancing as well. This is particularly true if they are required to monitor and enforce new working arrangements. Furthermore, the extent to which workers will have discretion to weigh virus-related risks in relation to the need to be present at work is not yet clear.

Workplace flexibility and modification will be needed to support safe workplace openings, and this will vary substantially by industry and occupation. Settings that involve working in close physical proximity to others will have elevated risk of infection (e.g., meatpacking), while in others, infection control measures are more feasible. The occupational rehabilitation literature shows that effective return to work programs are context specific [11], and it seems apparent that a similarly targeted approach will be required as workplaces re-open during the pandemic. Some workers may choose to work from home indefinitely or take extended leave from work. In some workplaces that pose untenable risks, it is possible that workers will seek other job roles or file for disability or unemployment payments.

## Societal Factors

The COVID-19 pandemic will have a long-term societal impact both in and out of work. Changes in social interaction will require that many standard practices within employing organizations be re-evaluated and revised. A substantial change in workplace interactions and work habits will require accommodation and leeway in workers with the most significant concerns, those with the greatest illness risks, and those who are working in the highest risk work environments. Just as with return-to-work after injury, employers may struggle to maintain uniform and fair practices while also being responsive to the concerns of individual workers, and it will be important to involve multiple stakeholders in this process [12].

The existing occupational rehabilitation literature has shown how return-to-work and other worker health and safety outcomes are stratified by income, language, immigration status, social rank, and other measures of socioeconomic advantage or disadvantage. Data from the COVID-19 pandemic will no doubt reflect that disadvantaged workers are overrepresented among essential workers and those deemed necessary for businesses to remain open or reopen.

Disadvantaged workers are at greatest risk of experiencing negative outcomes from COVID-19. Low-income workers will be more likely to have jobs deemed as essential with no options for working from home, and workplace exposures may be more difficult to control. Working from home may be impossible or impractical, and the threat of layoffs may lead low-income workers to accept workplace exposure hazards despite fears of infection and job loss. Other disadvantages related to age, race and ethnicity, language, education, or social status may result in fewer advantages for alternative work and job flexibility. Employer policies related to opening with COVID-19 will need to pay particular attention to consequences to workers who have socioeconomic disadvantages.

## Recommendations

The COVID-19 pandemic will challenge many of our existing conventional practices in occupational health and safety and work disability prevention, and the reopening process will see tremendous variation across workplaces and between workers in the same occupations. There is a complexity of multi-level factors that will influence whether individual workers will accept workplace safety risks, trust organizational measures and co-workers, modify work habits, return to shared working spaces, and resume productivity. Based on the lessons learned from the occupational rehabilitation literature, we recommend the following:

- Employer plans and strategies for reopening the workplace should identify and anticipate individual worker circumstances that will affect worker COVID-19 attitudes and behavior. Policies that demand uniform compliance may be unsustainable or unrealistic.
- Occupational health and safety guidelines for reopening the workplace should be industry- or occupation-specific and consider the unique physical, psychological, and social workplace factors of different work settings.
- Workplace openings should prioritize the needs of disadvantaged workers, as they are most likely to have higher environmental exposures and inflexible job tasks, and they will be most threatened by job loss and unemployment.

Successful opening of workplaces during the COVID-19 pandemic will require significant changes to organizational health and safety policies and practices to show flexibility to individual worker needs, to be fair to workers with less socioeconomic advantage, and to understand the backdrop of stress and social disruption being experienced at all levels of society.
